# Rift Valley Fever Vaccine Development, Progress and Constraints

**DOI:** 10.3201/eid1709.110506

**Published:** 2011-09

**Authors:** Jeroen Kortekaas, James Zingeser, Peter de Leeuw, Stephane de La Rocque, Hermann Unger, Rob J.M. Moormann

**Affiliations:** Author affiliations: Central Veterinary Institute of Wageningen University and Research Centre, Lelystad, the Netherlands (J. Kortekaas, R.J.M. Moormann);; Centers for Disease Control and Prevention, Atlanta, Georgia, USA (J. Zingeser);; Food and Agriculture Organization of the United Nations, Rome, Italy (J. Zingeser, P. de Leeuw, S. de La Rocque);; Centre International de Recherche Agronomique pour le Développement , Montpellier, France (S. de La Rocque);; International Atomic Energy Agency, Vienna, Austria (H. Unger)

**Keywords:** Rift Valley fever virus, workshop, vaccine, recommendations, viruses, conference summary

The workshop Rift Valley Fever Vaccine Development, Progress and Constraints was organized by the Food and Agriculture Organization of the United Nations (FAO) and the Central Veterinary Institute of Wageningen University and Research Centre, under the umbrella of the Global Framework for the Progressive Control of Transboundary Animal Diseases, a joint initiative of FAO and the World Organisation for Animal Health. The workshop was supported by the Netherlands Ministry of Economic Affairs, Agriculture and Innovation, and by the US Centers for Disease Control and Prevention; other participants included the World Health Organization and the International Atomic Energy Agency. The meeting occurred January 19–21, 2011, at FAO headquarters in Rome, Italy, and was attended by 34 leading scientists in Rift Valley fever virus (RVFV) vaccine development, representatives of international organizations, and policy makers. Stakeholders from industry were represented by the International Federation for Animal Health. The main objective of the meeting was to gain consensus about desired characteristics of novel veterinary RVFV vaccines and to discuss how incentives can be established to ensure that these vaccines come to market.

Historically, 2 vaccines have been available for control of RVFV in livestock. The first is based on the live-attenuated Smithburn virus (*1*). Although this vaccine is inexpensive and provides lasting immunity after 1 dose, its residual virulence renders it unsuitable for application in newborn and gestating livestock. A safe alternative is based on inactivated whole virus. For optimal immunity, however, this vaccine requires a booster and annual revaccination. Drawbacks of these classical vaccines explain the need for a new generation of RVFV vaccines.

Workshop participants agreed that novel vaccines should be cost-effective and should provide swift and long duration of immunity after a single vaccination and that application should be safe regardless of the physiologic state of the animal. The possibility of needle-free delivery would be advantageous, especially when absence of virus circulation cannot be definitely established and reuse of needles represents a risk for further dissemination. Novel vaccines that enable differentiation between infected and vaccinated animals (DIVA) by use of an appropriate discriminatory assay would be beneficial.

The live-attenuated candidate vaccines that were discussed during the meeting were the MP-12 vaccine (*2**–**6*), a recombinant RVFV that contains deletions in 2 of the 3 genome segments (*7*), and the clone 13 vaccine (*8**–**10*). Data presented during the workshop suggest that all 3 live-attenuated vaccine candidates are highly immunogenic and safe in ewes during the first trimester of gestation and that the MP-12 vaccine is immunogenic and a candidate for human vaccination. The clone 13 vaccine was recently registered and marketed in South Africa; the other live-attenuated vaccines could also come to market in the next decade. ELISAs based on nonstructural proteins could be used as DIVA assays to accompany these vaccines (*11*).

Alternative vaccines discussed during the workshop are based on the structural glycoproteins Gn and Gc. These proteins are presented by vaccine vectors, produced in vivo from plasmid (DNA vaccines), or administered in the form of virus-like particles (VLPs). Apart from the high safety profile of these vaccines, an additional advantage is their potential application as DIVA vaccines that can be accompanied by commercially available nucleocapsid protein–based ELISAs. A challenge for these approaches is development of a cost-effective vaccine capable of providing protection after 1 dose.

Vector vaccines discussed during the workshop are based on capripoxviruses, Newcastle disease virus (NDV), or modified vaccinia Ankara (MVA). It is hypothesized that multivalent capripoxvirus-based vector vaccines would be cost-effective, and their bivalent nature would make them attractive for inclusion in routine capripoxvirus immunization programs, thereby increasing immunity against RVFV. The experiments reported during the workshop suggest that capripoxvirus-vectored vaccines can provide protection against RVFV and capripoxviruses (*12**,**13*).

An alternative approach is based on a vaccine strain of NDV (*14*,*15*). Mammals are not natural host species of NDV, and the efficacy of NDV-based vector vaccines is therefore unlikely to be compromised by preexisting immunity in the field. Vaccination with an NDV recombinant expressing the RVFV structural glycoproteins Gn and Gc has protected mice from lethal challenge, and 1 dose given to lambs resulted in a neutralizing antibody response (*14*). MVA is also being evaluated as a vector of RVFV antigens. A single vaccination of mice with an MVA vector expressing Gn and Gc (MVA-M4) provided complete protection (A. Brun, unpub. data). MVA-M4 is not only a promising vaccine candidate for livestock but, considering its safety profile, may also be evaluated as a vaccine for humans.

Alternative vaccines with optimal safety profiles are alphavirus replicon–based vaccines (1*6*), DNA vaccines (*16**, **17*), and VLP-based vaccines (*18**,**19*). Initial vaccination with an alphavirus replicon–based vaccine followed by a booster has been shown to protect mice, and promising DNA vaccines based on RVFV genes fused with genes encoding molecular adjuvants have shown promise in mouse trials (*16**,**17*). Progress has also been made in approaches that use VLPs. To improve the stability, quantity, and uniformity of VLPs, the Gag protein of Moloney murine leukemia virus was added to VLPs, referred to as chimeric VLPs. Adjuvanted chimeric VLPs protected rats after a single vaccination (*18*). In an alternative approach, VLPs that express the nucleocapsid gene from a packaged minigenome were produced and provided complete protection in mice after a single vaccination (*19*). These results, together with recently established improved production methods, suggest that VLP-based vaccines can soon become cost-effective alternatives for live vaccines.

In conclusion, tremendous progress has been made in the development of novel vaccines for RVFV control. At the end of the workshop, participants drafted 11 recommendations to guide and facilitate the development of RVFV vaccines, norms and standards, and vaccine stockpiles for rapid deployment. These recommendations and other meeting documents are available at www.fao.org/ag/againfo/programmes/en/empres/RVF_2011.html.
